# Does Competency-Based Education Have a Role in Academic Pharmacy in the United States?

**DOI:** 10.3390/pharmacy5010013

**Published:** 2017-02-27

**Authors:** Melissa S. Medina

**Affiliations:** College of Pharmacy, The University of Oklahoma, 1110 N Stonewall Ave, Oklahoma City, OK 73117, USA; Melissa-medina@ouhsc.edu; Tel.: +1-405-271-6484

**Keywords:** competence, pharmacy, healthcare, program outcomes, education, standards

## Abstract

Competency-based Education (CBE) is an educational model that allows students to learn and demonstrate their abilities at their own pace. CBE is growing in popularity in undergraduate educational programs and its role in pharmacy education in the United States (US) is under review. In comparison, medical education is utilizing competency-based approaches (such as competencies and Entrustable Professional Activities) to ensure that students possess the required knowledge, skills, and attitudes prior to graduation or program completion. The concept of competency-based approaches is growing in use in pharmacy education in the US, but the future related to aspects of this concept (e.g., mandatory Entrustable Professional Activities) is not certain. A review of pharmacy education’s evolution in the US and a comparison of competency-related terms offers insight into the future use of competency-based approaches and CBE in pharmacy education in the US through the lens of benefits and challenges.

## 1. Introduction

In the United States (US), medical education has increased its interest in Competency-based Education (CBE) over the past several years, which has piqued interest in pharmacy. Formally, a CBE program is an educational model that removes traditional semester timeframes, allowing students to learn at their own pace and demonstrate what they know through assessments developed by the program [1]. Relatedly, competency-based approaches (including assessment of competencies) have been used in educational programs such as pharmacy, as seen in the 2016 Accreditation Council for Pharmacy Education (ACPE) Standards program outcomes which use the term competencies in relationship to outcomes [2,3]. It is important to note that a competency-based approach and competencies in pharmacy education are different than formal CBE, which removes semester timeframes. In order to understand the future of CBE in pharmacy education in the US, it is important to reflect on the past and present of pharmacy education; define the terminology related to CBE and competencies, and evaluate how other health professions (such as medical education) address CBE and competencies, which can offer insight into future directions for pharmacy education.

## 2. History of Pharmacy Education Standards

In the US in the 19th century, there was no legal requirement to learn the pharmacy profession through formal education and the apprenticeship model was the dominant training method [4]. State universities were the first to design formal pharmacist education models, starting in 1868 at the University of Michigan, where students enrolled in full-day courses over four terms (3-months long each) and no prior pharmacy work experience was required for admittance and in 1892, the University of Wisconsin established a four-year program [4]. During the 20th and 21st centuries, the Flexner report precipitated changes to the content and length of the pharmacy curriculum, mode of delivery, required prerequisites, and the degree earned [4,5]. There was also little uniformity in pharmacy licensure and no program accrediting bodies until 1932 when ACPE was founded [4].

**Figure 1:**
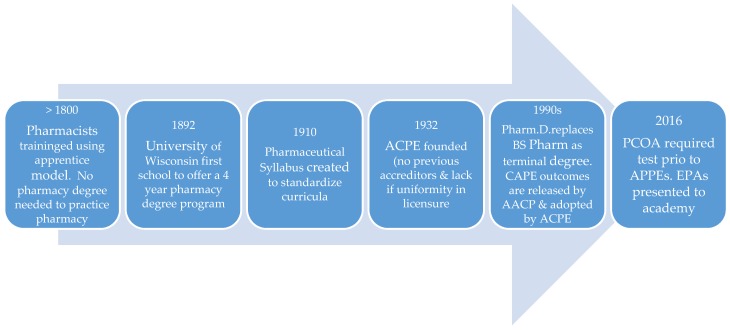
Timeline of Significant Pharmacy Curriculum Events in the US. ACPE = Accreditation Council for Pharmacy Education. AACP = American Association of Colleges of Pharmacy. CAPE = Center for the Advancement of Pharmacy Education outcomes; which are revised every 7 years (current version is 2013). PCOA = Pharmacy Curriculum Outcomes Assessment. APPE = Advanced Pharmacy Practice Experiences. EPA = Entrustable Professional Activities.

The US Department of Education (USDE) now recognizes ACPE as the organization that evaluates the quality of professional degree programs leading to the Doctor of Pharmacy degree, the standard entry level degree. To receive accreditation, Doctor of Pharmacy programs must meet expectations outlined in the 2016 ACPE Accreditation standards [2]. During the 20th and 21st centuries ACPE has overseen many changes in pharmacy education such as the length of the program from 4 to 6 years and the entry level degree from Bachelor of Science to Doctor of Pharmacy. Recently, major changes have occurred regarding how programs are delivered and there are now accelerated 3-year programs, online programs, and multi-site campuses that are connected through synchronous video-streaming. These changes to program delivery have resulted in changes to the accreditation standards, with the most recent update occurring in 2016 [2]. The reverse is also true, where changes in the accreditation standards have required changes to pharmacy curricula. The 2016 ACPE standards include emphasis on an affective domain (standard 4) based on the 2013 CAPE outcomes revision [2]. The growing importance of interprofessional education is seen in standard 11 [2] and the administration of the Pharmacy Curriculum Outcomes Assessment (PCOA) in the pre-advanced pharmacy practice experience (Pre-APPE) is delineated in standard 12 [2]. Standard 10 outlines Curriculum Design, Deliver, and Oversight requirements and states that the minimum curriculum duration is a minimum of four years of full–time study or the equivalent [2,6]. Standard 10.3 (knowledge application) and Standard 10.4 (skill development) indicate that students must demonstrate their competencies in both knowledge and skills and as a result, assessment of these competencies has grown in importance [2]. These significant events are outlined in Figure 1.

## 3. Definitions of Competency-Based Education (CBE) and Competency-Based Approaches

The growing importance of assessment has increased the terminology and concepts related to assessment. One of these newer concepts that has arisen in higher education is the term competency-based education. Higher education has historically used time (e.g., semesters and credit hours-formally known as the Carnegie Unit) as the yardstick for determining readiness, which arose in the early 1900s and formed the basis for program design, accreditation, and funding [1,7]. CBE in contrast emphasizes directly measuring how much students have learned (learning-based system) instead of how long they have spent learning (time–based system), which allows students to move at their own pace [1]. CBE programs are aimed at nontraditional students who need more flexible options to earn their first or second degree or update their skills [1]. These programs are more than just on-line programs because the focus instead turns to allowing students to demonstrate their achievement of required competencies which may have been gained during previous work experience, therefore allowing the more flexible awarding of credit in comparison to credit hours [7]. In CBE, students demonstrate mastery of explicit and measurable knowledge, skill, and attitude outcomes (competencies) and receive individualized support that is tailored to their specific developmental needs [7]. Students progress in the program by demonstrating they have mastered the knowledge and skills (competencies) for a course regardless of time, meaning they could take more or less time [8], therefore studying and learning at their own pace. CBE allows students to accelerate through what they already know and spend more time on what they do not know, which means students can accelerate (or delay) their progress toward a degree [8]. A comparison of traditional versus CBE can be seen in Table 1.

In comparison to CBE, which focuses on changing the structure and time requirements of educational programs, ultimately changing curricula, there are competency-based approaches that embed the teaching of competencies and assessment of competence into the existing curricula and traditional time-based structure [3]. Competency-based approaches are currently used in undergraduate and medical education and their use is growing in pharmacy education [3,9,10]. Therefore, the future of competency-based approaches is now. Within this approach, there are competencies, which are predefined abilities or outcomes of a curriculum [10]. There is also competence that can be thought of as progression toward professional expertise or demonstration of a predefined skill or knowledge level that is multi-dimensional, dynamic, contextual, and developmental [10]. Competencies describe qualities of professionals and measuring professional competence can be difficult [11]. One way that medicine has evaluated competencies of their students or trainees within the medical curricula is to use Entrustable Professional Activities (EPAs) [12] and pharmacy education has focused recent attention on EPAs as well [3]. The terms EPA and competencies should not be used interchangeably because EPAs are descriptors of work and translate competencies in professional practice whereas competencies describe physicians [11,12]. Outlining core EPAs is a way to ensure that students are practice ready upon graduation [3] which is an aim of the 2016 ACPE Accreditation Standards [2]. EPAs reflect the level of supervision required for students (e.g., direct vs. distant supervision) and are aimed at establishing the level of proficiency that is required for professional practice upon completion of training or graduation [11]. When an EPA is first learned and practiced, the level of supervision needed may be high, which would be considered developmentally appropriate and expected for early leaners [3,11].

Competency-based approaches as described above are currently in use and development. In the future, although the EPAs are not officially required in the ACPE standards 2016, it is possible they will follow the path of the CAPE Outcomes and become adopted in the standards [2,6]. It is also possible that in the future, EPAs may set the stage for required mandatory skills-based examinations (such as Objective Structured Clinical Exams), similar to the PCOA exam The ACPE standards 2016 have become more prescriptive in this version related to assessment as a way for programs to increase their transparency while working on continuous quality improvement [2]. Key elements in Section 3 require formative and summative assessments as well as mandatory, standardized, and comparative assessments [2]. This section also discusses student achievement and readiness to “enter APPE, provide direct patient care in a variety of settings, and contribute to an Interprofessional collaborative patient care team” [2] (p. 25). The assessment standards offer colleges and schools of pharmacy more guidance on how they should demonstrate that their students have learned and achieved the educational outcomes and as a result, an OSCE-like exam based on competencies and EPAs is possible.

While competency-based approaches are emerging in current pharmacy curricula with attention on EPAs, the appeal is that the competency based assessment can provide a mechanism to prevent students from graduating from a pharmacy program unless they have demonstrated the predefined and expected level of competence for program outcomes [3]. This appeal is a subtle yet important distinction because in its current and near future use EPAs require students to demonstrate and achieve OR remediate deficient knowledge and skills prior to graduation within the existing curricular structure. Students can take more time if needed but it must be completed within the allotted timeframe and academic standing policies. EPAs do not currently allow an open-ended and limitless timeframe. Although competency-based approaches are used in medicine and pharmacy, it is unclear what the future holds for formal CBE. There are benefits and challenges to the design.

## 4. Benefits and Challenges of CBE in Pharmacy Education

Frank and colleagues [10] described benefits to medical education and these benefits can be extrapolated to pharmacy education. (1) Defines consistent competencies and milestones. CBE would help pharmacy educators define competencies expected of graduates and developmental milestones prior to graduation, better ensuring that all students possess the same level of baseline skills upon graduation; (2) Determines acceptable levels of performance for competencies and milestones. CBE would promote a national discussion of what constitutes an acceptable level of evidence of abilities; such as when are students expected to demonstrate novice, competent, proficient, or expert performance for specific competencies. This would better align faculty expectations so that one faculty member does not expect a higher or lower level than another faculty member; (3) Outlines acceptable assessment methods and tools for assessing the competencies. CBE would shape what assessments best measure the outcomes of specific competencies. It would also better ensure that assessment of graduates’ abilities would not vary as a result of programmatic, regional, or local differences; (4) Offers flexibility in learning. CBE would offer students a more flexible timeframe to demonstrate competencies and therefore allow them to progress at their own rate, which is more learner centered and personalized [10].

There are challenges associated with CBE in pharmacy education which can be inferred from medicine [10]. (1) Presents IPPE and APPE logistical concerns. The biggest challenge to using CBE is that moving students through time-based curricula is efficient and manageable. For example, it is unclear how programs will accommodate students on introductory and advanced pharmacy practice experiences (IPPE and APPE) when the prescribed number of weeks is removed but preceptor laws remain and some sites can only accommodate a limited number of students; (2) Complicates faculty time allocation. When students complete course content at different times, it is unclear how faculty would handle assessment of knowledge, skills, and attitudes in an efficient manner. There is an efficiency to administering exams to an entire class during a set time block. It is possible that faculty would spend a majority of their time assessing knowledge, skills, and attitudes on an individual basis for the didactic portion of the program, leaving little time to teach and assess on IPPE and APPEs as well as fulfill other parts of the tripartite mission; (3) Makes managing poor student performance and progression difficult. Pharmacy curricula are designed to have courses and content build upon each other. While students can self-pace, much of the course work is lock-step in nature. It is not clear how programs will manage students completing prescriptive course work at different rates. In addition, many programs have some rate of attrition due to poor performance. Academic standing committees would need to establish time limits and maximum number of attempts for students to complete competencies, which could be logistically difficult to manage. CBE is also less structured by design, which may lead to more student dismissals as a result of weak students who may not manage their time well. The structure offered in time-based curricula can benefit academically at-risk students, whereas the lack of structure in CBE may hurt that category of students; (4) Creates a narrow focus of curricula. A focus on completing competencies can shift attention from the big picture of how content within a curriculum builds up and advances to a more fragmented picture of small units of performance and “jumping through hoops” which can frustrate faculty and students [10]. Focus can also shift from learning goals to performance goals, which are indicative of a fixed mindset where students are more likely to cheat, give up when faced with failure, and focus on receiving validation from others instead of striving for competence and mastery [13,14]; (5) Shifts attention from knowledge to skills. Previous complaints have arisen that pharmacy is too content heavy and that students may enter professional practice lacking skills. Shifting to CBE may create an imbalance in the opposite direction where skills are more valued than knowledge, emphasizing the role of the pharmacist as a technician versus a health-care provider and problem-solver.

## 5. Discussion

Overall, CBE is an instructional model that is built on eliminating time-based curricula. Based on this definition, the use of CBE in US pharmacy education is unclear. A review of the literature suggests the CBE definition is applied broadly and the future of the concept competency-based approaches (e.g., EPAs) where attention is placed on students demonstrating competencies during the traditional time-limited and structured program is currently being implemented and grown in pharmacy education. There are still areas of future uncertainty related to competency-based approaches such as mandatory EPAs and required national OSCE assessments in ACPE program accreditation. The future of formal CBE in pharmacy education has benefits and challenges. CBE appears to be difficult to implement, especially in a political climate where colleges and universities are asked to do more with less money and resources. While the pharmacy academy may benefit from ensuring that students can meet specific competencies at predefined levels along the expert-novice continuum, removing time-based curricula may not be feasible in the immediate future.

## Figures and Tables

**Table 1:** Comparison of traditional vs. competency based education.

Curricular Concept	Traditional Instruction	CBE
**Structure**	Time-based, semesters and credit hours	Learner-centered; Competency-based
**Teaching mode**	Group learning, emphasis on knowledge	Individualized, tailored, emphasis on abilities or competencies
**Pace**	Faculty-paced; all students move together through content at same time; structured	Self-paced; movement through content determined by individual student’s competency attainment; flexible
**Assessment method**	Summative, high stakes	Mastery-learning, performance-based
**Program completion time**	Finish when all required courses are passed	Finish when mastery of competencies demonstrated
